# *Streptococcus mitis* Chorioamnionitis after Dental Scaling and Oral Sex

**DOI:** 10.1155/2020/9251731

**Published:** 2020-11-02

**Authors:** Boshra Sara Hosseini, Jennifer Hunt

**Affiliations:** Department of Obstetrics, Gynaecology and Reproductive Sciences, University of Manitoba, Winnipeg, Manitoba, Canada

## Abstract

**Background:**

Oral sex is postulated to be a risk factor for the introduction of bacteria into the amniotic cavity. Common oropharyngeal bacteria have been implicated in reports of second trimester chorioamnionitis via ascending vaginal transmission following oral sex. Dental scaling can also introduce these pathogens into the blood stream, allowing hematogenous spread of oral pathogens to the fetoplacental unit in pregnant patients.

**Case:**

We report a case of *Streptococcus mitis* chorioamnionitis at 21 weeks and 5 days' gestation in a patient whose only risk factors were recent dental scaling and recent oral sex with a partner known to have periodontal disease.

**Conclusion:**

Bacterial chorioamnionitis should be considered in the differential diagnosis of preterm labour. Oral sex and dental procedures may be risk factors for chorioamnionitis.

## 1. Introduction

*Streptococcus mitis* (*S. mitis*), a viridans group streptococcus, is a normal microflora of the human oropharynx. For the majority of people, *S. mitis* has a benign presence. However, it can cause a variety of infections ranging from dental caries to bacterial infective endocarditis, bacteremia, meningitis, eye infections, and pneumonia [1].

The presence of *S. mitis* in chorioamnionitis has been reported infrequently in the literature. Hematogenous spread, iatrogenic introduction through amniocentesis, and ascending vaginal transmission have all been postulated as potential mechanisms. There is only one other case report that has demonstrated an association between oral sex, chorioamnionitis, and viridans streptococcal species isolated from amniotic fluid obtained by amniocentesis [2]. In this case, cultures of the fetal and maternal surfaces of the placenta were not done and the placenta was not submitted for pathology.

Other oral bacteria have also been implicated in chorioamnionitis. Four such cases suggested a close temporal relationship between receptive oral sex and chorioamnionitis [3–6]. Although hematogenous spread is plausible, there have not been any reported cases of chorioamnionitis caused by oral bacteria after dental scaling.

## 2. Case

A 43-year-old female, gravida 4 para 3, presented in active preterm labour at 21 weeks and 5 days' gestation. Her cervix was 4-5 cm dilated with the amniotic sac bulging into the vagina. Membranes were not ruptured. She was afebrile with no evidence of any infectious symptoms.

She delivered a male infant approximately 9 hours after the onset of contractions. The infant weighed 510 grams and died approximately 1 hour after delivery. A neonatologist assessed the infant postpartum and determined that it was consistent with a gestational age of approximately 22 weeks.

Her prenatal history was unremarkable. She had three normal ultrasounds within the pregnancy: a normal dating ultrasound at 7 weeks' gestation, a normal nuchal translucency of 1.0 mm at 12 weeks' gestation, and normal fetal anatomy at 20 weeks' gestation. Cervical length was normal on sonographic images. In addition, chorionic villus sampling was performed at 12 weeks and 5 days' gestation, and a normal male karyotype (46XY) was obtained. The patient had had routine dental scaling performed 14 days prior to delivery. She did not have any history of gingival disease or cavities but had been noticing malodorous breath. Her husband had known periodontal disease, and the couple had engaged in oral sex approximately 10 days prior to delivery.

A culture of the amniotic membrane was performed that isolated *S. mitis*. Placental pathology showed signs of acute chorioamnionitis including acute inflammation of the placental plate chorion and acute funisitis of the three-vessel umbilical cord.

## 3. Discussion

Viridans group streptococci have been implicated as a cause of preterm labour, intrauterine fetal demise, neonatal sepsis, and meningitis [2]. S*. mitis*, a member of viridans group streptococci, is a gram-positive facultative anaerobe and catalase negative bacteria that is part of the oropharyngeal microflora. It uses a variety of strategies to effectively colonize the human oropharynx. These include modulation of the host immune system and expression of adhesins, immunoglobulin A proteases, and toxins. These strategies can act as virulence factors that may promote opportunistic infections [1].

The presence of *S. mitis* in the amniotic fluid is rare and suggests hematogenous, ascending, or iatrogenic transmission. To our knowledge, there have been two previous reports of *S. mitis* chorioamnionitis. Waites et al. reported a case of *S. mitis* isolated from amniotic fluid obtained by amniocentesis at 16 weeks' gestation in a patient who developed signs of chorioamnionitis and a subsequent stillbirth [7]. Schmiedel et al. described a second case in which *S. mitis* was isolated from intraoperative swabs taken from fetal membranes in a patient with fulminant chorioamnionitis. When the samples were analyzed with fluorescent *in situ* hybridization, *S. mitis* was only isolated from the superficial layers of fetal membranes and was not present within the placental tissue itself [8]. This supports an ascending route of infection, postulated to be more likely following cunnilingus.

Oral hygiene is the strongest predictor of gum disease, but there are other risk factors caused by differences in plaque microflora and host immune function. Pregnancy, oral contraceptive use, smoking, and diabetes have been associated with increased susceptibility to gum disease [9]. Harville et al. found that engaging in oral sex was associated with gum disease [10]. The authors suggested that oral sex might introduce microbes and/or mechanical trauma into the oral cavity. It is also possible that oral sex might spread infection from the oral cavity to the genital tract.

The safety of oral sex in pregnancy is not known. There have been several reports of life-threatening complications following oral sex during pregnancy, such as venous air embolism. Chorioamnionitis caused by common oropharyngeal bacteria is another possible complication although it is reported in only five previous cases in the literature [2–6]. Our patient did present in a similar manner to the patients described in these instances. In each case, women presented in preterm labour between 21 and 26 weeks' gestation. All patients admitted to engaging in oral sex between two weeks and one day before the onset of symptoms. Common oropharyngeal bacteria such as *Fusobacterium nucleatum*, *Capnocytophaga* sp., *Eikenella corrodens*, and viridans streptococcal species were isolated from amniotic fluid or placental cultures. In at least two of the five cases, poor periodontal health was noted in the partner.

Hematogenous transmission of *S. mitis* is also possible and has been reported after dental procedures [11]. Periodontal diseases are also regarded as a risk factor for adverse pregnancy outcomes including gestational diabetes, preeclampsia, preterm birth, and low birth weight. It has been postulated that pathogenic oral bacteria originating from the gingival biofilm may directly infect the placenta subsequent to bacteremia or that inflammatory mediators secreted from the gingival mucosal surfaces are carried to the placenta where they trigger an inflammatory response [12]. Even though our patient admitted to good oral health, it is possible that dental scaling performed 14 days prior may have contributed to the resulting infection.

We highlight that she was asymptomatic at the time of presentation with no evidence of maternal infection. Subclinical chorioamnionitis on placental pathology was evidence for our proposed theory that *S. mitis* was acquired either as a result of seeding from her oral cavity after dental scaling or as a result of an ascending infection related to receptive oral sex. To date, we have not found another case within the literature of *S. mitis* chorioamnionitis after dental scaling.

Preexisting colonization of the vagina with *S. mitis* could also lead to an ascending infection and subsequent chorioamnionitis. Although we are unable to exclude this possibility in our patient, *S. mitis* is not part of the normal vaginal flora. It is plausible that she could have acquired the pathogen if she engaged in regular oral sex with her partner known to have active periodontal disease.

Bacterial colonization of the amniotic fluid should be considered in patients presenting with preterm labour. A history of recent oral sex and dental scaling may be associated with chorioamnionitis. It is reasonable to explore these risk factors in the history of a patient presenting in preterm labour without any other etiology. The safety of oral sex in pregnancy cannot be evaluated due to the small number of reported cases of complications.
